# The Role of Telemedicine in Emergency Department Triage and Patient Care: A Systematic Review

**DOI:** 10.7759/cureus.75505

**Published:** 2024-12-10

**Authors:** Anas A Ahmed, Mohammed E Mojiri, Ali A Daghriri, Ohoud A Hakami, Reem F Alruwaili, Rayan A Khan, Hassan A Madkhali, Manar M Almania, Zaher T Hakami, Khadijah O Mashraqi, Khowlah A Adawi, Sadeel A Alqattan, Ahmad N Alharbi, Malek A Albahlol, Atheer I Moafa

**Affiliations:** 1 Community Medicine, Jazan University, Jazan, SAU; 2 College of Medicine, Jazan University, Jazan, SAU; 3 College of Medicine, Al Jouf University, Sakakah, SAU; 4 College of Medicine, Ibn Sina National College for Medical Studies, Jeddah, SAU; 5 College of Pharmacy, Jazan University, Jazan, SAU; 6 College of Medicine, Majmaah University, Al Majma'ah, SAU; 7 College of Medicine, Arabian Gulf University, Manama, BHR; 8 College of Medicine, King Abdulaziz University, Jeddah, SAU; 9 College of Medicine, King Saud University, Riyadh, SAU

**Keywords:** diagnostic accuracy, emergency department, healthcare efficiency, patient care, patient satisfaction, remote consultation, systematic review, telemedicine, triage

## Abstract

Overcrowding in emergency departments (EDs) is a global challenge, leading to prolonged waiting times and adverse patient outcomes. Telemedicine has emerged as a promising solution, enabling remote consultation, triage, and real-time specialist input. Despite its growing application, limited systematic research exists on its specific role in ED triage and care. This systematic review evaluated the effectiveness of telemedicine interventions in ED settings, focusing on diagnostic accuracy, patient satisfaction, throughput times, and re-admission rates. Following Preferred Reporting Items for Systematic Reviews and Meta-Analyses (PRISMA) guidelines, a comprehensive search of PubMed, Web of Science, Scopus, and the Cochrane Central Register of Controlled Trials (CENTRAL) databases was conducted up to November 10, 2024. Eligible studies were randomized controlled trials (RCTs) in English that assessed telemedicine in ED settings. Two independent reviewers screened studies, extracted data, and assessed methodological quality using the modified Downs and Black scale. Six RCTs met the inclusion criteria. Results showed that telemedicine improved diagnostic accuracy, reduced re-consultation rates, and enhanced treatment adherence. High diagnostic concordance with in-person assessments was observed, particularly in non-critical cases. Several studies reported shorter throughput times and higher patient satisfaction with telemedicine compared to traditional care. However, telemedicine's impact on high-acuity cases, such as emergency airway management, was less pronounced. Limitations included small sample sizes and the inability to blind participants, which may have influenced outcomes. Telemedicine holds significant potential to improve ED triage and care in non-critical cases, reducing waiting times and enhancing patient outcomes. Further research is needed to establish its efficacy in high-acuity settings and address challenges related to infrastructure and privacy. Telemedicine should complement in-person care as part of an integrated ED strategy.

## Introduction and background

The global burden on emergency departments (EDs) continues to rise, driven by increasing patient numbers, resource limitations, and the complexity of cases presenting to acute care settings. Overcrowding in EDs is a well-documented issue, leading to prolonged waiting times, delayed treatments, and, in some cases, adverse outcomes for patients [1]. The introduction of telemedicine in this context offers a potential solution by facilitating remote consultations, triaging patients before they arrive at the ED, and enabling real-time specialist input for complex cases [2]. This approach may reduce unnecessary ED visits while ensuring that patients receive appropriate care in a timely manner.

Telemedicine has been used in various forms, such as real-time video consultations, store-and-forward systems, and tele-triage platforms [3]. In the ED setting, these technologies have shown promise in providing remote specialist consultations, monitoring critical patients, and supporting decision-making processes for triage. For instance, video consultations allow emergency physicians to collaborate with off-site specialists, improving diagnostic accuracy and reducing unnecessary transfers [4]. Similarly, tele-triage systems help prioritize patients based on the severity of their conditions, ensuring that those who need immediate care are identified early, even before they arrive at the hospital [5].

The COVID-19 pandemic has further accelerated the adoption of telemedicine, particularly in emergency settings, where minimizing physical interactions became a priority to reduce the spread of the virus. During the pandemic, telemedicine was employed to screen patients remotely, assess symptoms, and provide guidance on whether a physical visit to the ED was necessary [6]. This not only helped in managing patient volumes but also ensured that hospitals could allocate resources more efficiently while minimizing the risk of infection to healthcare workers and other patients. The pandemic has thus highlighted the critical role of telemedicine in enhancing the resilience of healthcare systems during public health emergencies [7].

Despite the growing interest in telemedicine for ED care, challenges remain regarding its implementation and effectiveness. Concerns about the reliability of remote diagnoses, the adequacy of technological infrastructure, and varying levels of user acceptance among healthcare providers and patients have been raised [8]. Furthermore, the legal and regulatory frameworks governing telemedicine, particularly regarding patient privacy and data security, continue to evolve [9]. These factors underline the need for comprehensive evaluations of telemedicine’s role in emergency care to ensure that its benefits are maximized while addressing potential drawbacks.

Previous systematic reviews have explored telemedicine in various healthcare settings, but few have focused specifically on its use in EDs. This review addresses this gap by synthesizing available evidence on the effectiveness, safety, and patient outcomes associated with telemedicine interventions in ED triage and care. It explores key outcomes such as diagnostic accuracy, patient satisfaction, throughput times, and the impact on re-admission rates [10].

## Review

Methods

Literature Search Strategy

This study adhered to the Preferred Reporting Items for Systematic Reviews and Meta-Analyses (PRISMA) guidelines during the conduct and reporting of this review [10]. Our search strategy included four major online databases: PubMed, Web of Science (WOS), Scopus, and the Cochrane Central Register of Controlled Trials (CENTRAL), covering the period from inception until November 10, 2024. Specific keywords such as "telemedicine, virtual medicine, telehealth, eHealth, telecare, emergency department, emergency room, and ER" were combined using Boolean operators. The search strategy was tailored to each database, and filters were applied to include only English articles involving human participants. Additionally, we manually examined the reference lists of included studies to identify any relevant articles that may have been missed during the initial search process.

Eligibility Criteria

Selection criteria were established using the PIOCS framework (P-population, I-intervention, C-comparison, O-outcome, S-study design). We included only English randomized clinical trials that included patients with various conditions; utilized telehealth either alone or as adjuvant therapy; compared telehealth to placebo, no intervention, or other therapies; and measured any outcome to assess the intervention's effect. We excluded observational studies, studies published in languages other than English, and published abstracts without full-text articles.

Study Selection

Two reviewers independently screened the titles and abstracts of retrieved articles based on predetermined eligibility criteria. Discrepancies or disagreements were resolved by a third reviewer until a consensus was reached.

Data Extraction

The full text of the included articles was analyzed, and the following data were extracted: country of research, participant age, sample size, type of telehealth intervention, patient condition, outcome measures, and main results. Any conflicts during data extraction were resolved by a third reviewer.

Quality Appraisal

The methodological quality of the included studies was assessed independently by two reviewers using the modified Downs and Black scale for clinical trials [11]. This scale includes 27 questions evaluating four categories: (1) reporting, (2) external validity, (3) internal validity, and (4) power. Studies scoring between 26 and 28 were considered excellent, 20 to 25 good, 15 to 19 fair, and 14 or lower poor. Any discrepancies in scoring were resolved through discussion until a consensus was achieved.

Data Synthesis and Analysis

A meta-analysis was conducted if at least two studies compared the efficacy of telehealth interventions on the same outcome. Standardized mean difference (SMD), 95% confidence interval (CI), and P-values were calculated to compare changes in outcomes between telehealth and control groups using a random-effects model [12]. Heterogeneity in treatment effects was assessed using the I² index. Statistical significance was set at P ≤ 0.05. All meta-analyses were performed using the Comprehensive Meta-Analysis software, version 2.2.064 (Biostat, Englewood, New Jersey, USA).

Results

Study Selection

**Figure 1:**
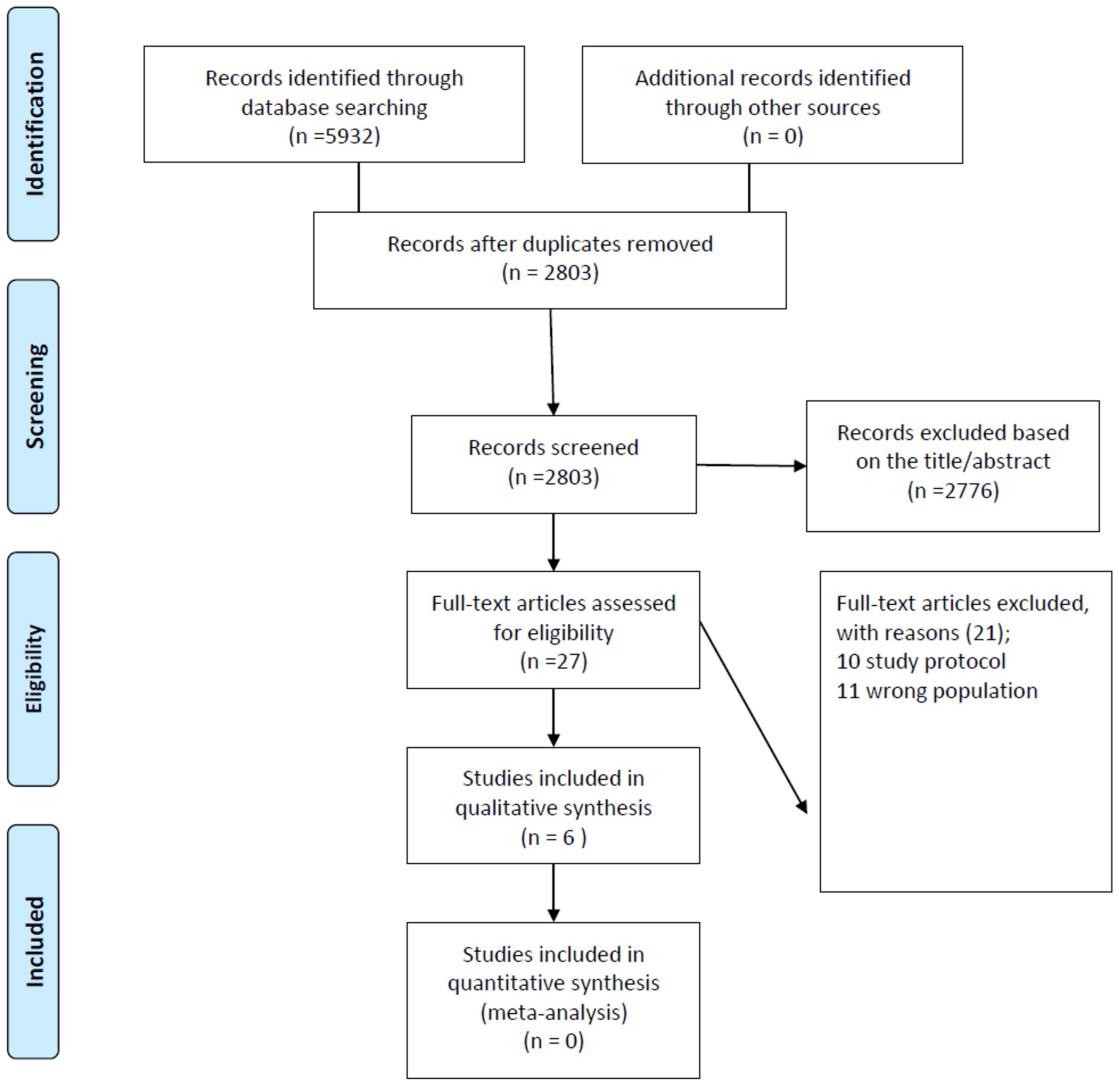
PRISMA flowchart of study search and selection PRISMA: Preferred Reporting Items for Systematic Reviews and Meta-Analyses

A total of 5,932 records were identified through database searching during the initial literature review. No additional records were found through manual searches of reference lists or grey literature. After removing duplicates, 2,803 unique records remained for screening. Titles and abstracts of these records were reviewed, resulting in the exclusion of 2,776 records that did not meet the inclusion criteria or were deemed irrelevant to the research question (Figure 1).

Following title and abstract screening, 27 full-text articles were retrieved for further eligibility assessment. Of these, 21 articles were excluded for various reasons, including issues with study design or protocol (11 articles) and irrelevance or a focus on the wrong population (10 articles). Ultimately, six studies were included in the qualitative synthesis for narrative review or descriptive analysis [12-17]. However, none of the studies were included in the quantitative synthesis (meta-analysis), as their data were unsuitable for such analysis or the review focused exclusively on qualitative data.

Study Characteristics

All included studies employed randomized controlled trial (RCT) designs conducted across various countries, demonstrating global interest in telemedicine as an alternative to traditional patient care (Table 1). The studies were conducted in Brazil [17], Tunisia [14], the USA [13], Korea [16], Switzerland [15], and Germany [12].

**Table 1:** Baseline characteristics of included studies RCT: randomized controlled trial; E1: experimental group 1; CG: control group; COVID-19: coronavirus disease 2019; COPD: chronic obstructive pulmonary disease; TM: telemedicine; HIPAA: Health Insurance Portability and Accountability Act; InTouch Health: a telehealth platform (brand name); FRED: friendly roll-about engineered for doctors; TAMS: Tele-Airway Management System; Gbit/s: gigabit per second; SMASS: Swiss Medical Assessment System; ESI: Emergency Severity Index; PC: personal computer

First Author, Year of Publication	Country	Design	Patient Conditions	Sample Size	Age (Years)	Telemedicine Type, Device, and Technology Used
Accorsi, 2022 [17]	Brazil	RCT	Acute respiratory tract infections during COVID-19 pandemic	98; E1 (48), CG (50)	36.3 ± 9.7	Telemedicine type: Direct-to-consumer TM; Device: Digital tablet (Apple iPad); Technology: Licensed HIPAA-compliant platform (InTouch Health), connected to institutional wireless network, using Cerner Millennium electronic records system
Soltane, 2024 [14]	Tunisia	RCT	Non-emergency conditions such as benign infectious diseases, trauma, mild decompensation of chronic conditions (asthma, COPD, heart failure), and suspected COVID-19 cases	400; E1 (200), CG (200)	E1 (45.10 ± 17.93), CG (42.73 ± 17.96)	Telemedicine type: Telephone follow-up; Device: Standard hospital telephone (no specific telemedicine platform used)
Brennan et al., 1999 [13]	USA	RCT	Non-emergency complaints in an emergency department (e.g., abrasions, allergic reactions, eye conditions, etc.)	104; E1 (54), CG (50)	Not specified	Telemedicine type: Real-time telemedicine using video workstations ("Friendly Roll-about Engineered for Doctors" or FRED), with peripherals such as digital stethoscope, otoscope, dermascope. 1.5 Mbit/s digital circuit for connection.
Cho et al., 2011 [16]	Korea	RCT	Emergency airway management (patients requiring intubation)	25; E1 (12), CG (13)	E1 (47 ± 19), CG (53 ± 19)	Telemedicine type: Tele-Airway Management System (TAMS) using a 1 Gbit/s intranet connection, two video cameras, and a video laryngoscope. The system provides remote guidance for intubation using real-time video and vital sign monitoring.
Meer, 2024 [15]	Switzerland	RCT	Patients visiting a walk-in clinic or emergency department; Emergency Severity Index (ESI 1-5)	2543 patients total	18-49 (54.94%), 50-65 (26.27%), 66-80 (14.16%), >80 (4.64%)	Telemedicine type: Telemedicine self-triage tool; SMASS (Swiss Medical Assessment System) pathfinder; Web-based symptom-checker utilizing computerized neural network
Villa, 2020 [12]	Germany	RCT	Dermatological emergencies in the emergency department	100; E1 (50), CG (50)	42.84 ± 18.72	Telemedicine type: Store-and-forward teledermatology; Device: Tablet PC (Pokini TAB A8, EXTRA Computer GmbH); Technology: Photographic acquisition and transmission of skin lesions for tele-evaluation

Each study addressed specific healthcare needs. For instance, one study examined acute respiratory infections during the COVID-19 pandemic [17], while another focused on benign infectious diseases, trauma, mild chronic condition decompensation, and suspected COVID-19 cases [14]. One study investigated non-emergency complaints in an ED, such as abrasions and allergic reactions [13], and another explored emergency airway management for patients requiring intubation [16]. One trial studied telemedicine self-triage tools in walk-in clinics and EDs [15], while another focused on dermatological emergencies in an ED [12].

Sample sizes varied significantly across the studies. One trial had the smallest sample size (25 patients) [16], while another had the largest (2,543 patients) [15]. Age distributions also varied, with one study involving a younger population (mean age of 36.3 years) [17], while another included patients with broader age ranges [14].

The technologies and devices used for telemedicine interventions differed across studies. One study used a Health Insurance Portability and Accountability Act (HIPAA)-compliant digital tablet platform integrated with the hospital’s electronic medical records [17]. Another employed a simpler telephone follow-up system without a specific telemedicine platform [14]. Real-time video workstations and peripheral devices were used in one study [13], while another utilized an advanced Tele-Airway Management System [16]. A telemedicine self-triage tool was deployed in one study [15], and a store-and-forward teledermatology system was employed in another [12].

Quality Assessment

Most studies performed well in reporting, with three studies scoring 10/10 [12,15,17]. Others scored slightly lower due to incomplete reporting of some patient characteristics [13,16]. External validity scores ranged from 2/3 to 3/3, with some studies limited by their specific settings, while others demonstrated broader applicability (Table 2).

**Table 2:** Quality of the included studies This table presents the quality assessment of the included studies based on the criteria outlined by Downs and Black. The studies were evaluated for the following risk factors: reporting, external validity, internal validity (bias), internal validity (confounding), and power. The total score for each study was calculated by summing the scores across these factors, with higher scores indicating better study quality. The risk of bias was assessed based on factors such as the adequacy of randomization, allocation concealment, blinding, and completeness of follow-up.

First Author, Year	Reporting (10)	External Validity (3)	Internal Validity - Bias (7)	Internal Validity - Confounding (6)	Power (1)	Total Score
Accorsi, 2022 [17]	10	2	5	5	0	22
Soltane, 2024 [14]	9	2	3	4	1	19
Brennan, 1999 [13]	8	2	3	3	1	17
Cho, 2011 [16]	8	2	2	3	0	15
Meer, 2024 [15]	9	3	5	5	1	23
Villa, 2020 [12]	9	3	3	5	0	20

Blinding was a common challenge, particularly for telemedicine interventions. Several studies could not blind participants or assessors [12,14,17], resulting in lower scores. Control of confounding factors varied across studies, with some adjusting for demographic variables such as age and gender. Statistical power was adequately addressed in some studies, while others lacked power calculations or had small sample sizes, limiting their ability to detect significant effects.

Effect of Interventions

Studies showed that telemedicine diagnostic accuracy was comparable to face-to-face clinical assessments. One study reported moderate agreement (Kappa = 0.386, p = 0.536) [17], while others reported high diagnostic concordance (Table 3) [12,15].

**Table 3:** Outcomes of the included studies EG: experimental group; CG: control group; TM: telemedicine; ED: emergency department; URTI: upper respiratory tract infection; ICD-10: International Classification of Diseases, 10th Revision; RT-PCR: reverse transcription polymerase chain reaction; Kappa: Cohen's Kappa (a statistical measure of inter-rater agreement); Re-co: re-consultation; TAMS: Tele-Airway Management System; OSD: on-scene directed; SMASS: symptom-checker for medical assistance and symptom support; WIC: walk-in clinic; PC: personal computer; p: probability value (used in statistical tests); mins: minutes

First Author, Year	Intervention of EG1 and How the Telemedicine Was Conducted in Detail	Intervention of CG and How the Conventional Treatment Was Conducted in Detail	Outcome Measures	Results
Accorsi, 2022 [17]	Patients received a brief telemedicine consultation before face-to-face evaluation. The diagnosis was based on the patient's history and institutional protocols, and telemedicine physicians could consult a decision support system integrated into the electronic medical records.	Patients in the control group (ED Group) received direct face-to-face evaluation at the emergency department (ED) without a telemedicine consultation.	• Final diagnosis based on International Classification of Diseases (ICD-10) codes. • Length of stay in the emergency department, tests ordered. • Medical prescriptions.	The study found that telemedicine was not inferior to face-to-face consultations in diagnosing acute respiratory tract infections, with telemedicine diagnosing 67.4% of patients with upper respiratory tract infection (URTI) compared to 72.1% in face-to-face evaluation (Kappa = 0.386, p = 0.536). COVID-19 RT-PCR test ordering rates were similar, with 76.5% in the telemedicine group and 79.4% in the face-to-face group (Kappa = 0.715, p > 0.999). Telemedicine also tended to prescribe fewer antibiotics (5.9% vs. 17.6%), though this difference was not statistically significant (Kappa = 0.452, p = 0.125). The average emergency department length of stay was similar in both groups (80.8 minutes for telemedicine vs. 97.9 minutes for face-to-face, p = 0.167), indicating telemedicine as an effective alternative for managing low-risk patients.
Soltane, 2024 [14]	The telemedicine group received follow-up at home via structured telephone calls on days 2, 7, 15, and 30 after discharge from the emergency department. Interventions included adjusting treatment, changing dosage, stopping treatment, or referring patients to specialists.	The control group received standard care with only a follow-up phone call on day 30 after discharge from the emergency department.	• Re-consultation rate and treatment adherence. • Patient satisfaction.	Re-consultation rate: Telemedicine group had significantly fewer re-consultations within 30 days (14%) compared to the control group (26.5%) (p = 0.004). Treatment adherence: Higher in the telemedicine group (97.5%) versus the control group (92%) (p = 0.014). Patient satisfaction: Higher in the telemedicine group (90%) compared to control (37.5%).
Brennan, 1999 [13]	Telemedicine group: Patients were evaluated by a telemedicine nurse at the suburban (base) hospital and treated by a remote emergency physician located at a rural hospital using a telemedicine link.	Patients were directly evaluated and treated in-person by an emergency physician at the suburban hospital (base site).	• Throughput time (time from admission to discharge), 72-hour return visits, need for additional care. • Satisfaction of patients, nurses, and physicians.	Throughput time: Telemedicine group had a shorter average throughput time (106 minutes) compared to the control group (117 minutes). 72-hour return visits: No significant difference between groups (0%). Need for additional care: No significant difference. Patient satisfaction: High in both groups, with telemedicine patients slightly more satisfied with the care received.
Cho, 2011 [16]	Tele-Airway Management System (TAMS): Remote guidance for emergency airway management using two video cameras, a video laryngoscope, and patient monitor. Intubation was performed by emergency residents under remote supervision from an emergency physician at a different hospital.	On-Scene Directed (OSD): Intubation was performed by emergency residents under the direct supervision of an emergency physician on site.	• Intubation time (time taken from picking up the laryngoscope to confirming tube placement). • Rate of intubation, complications (e.g., esophageal intubation).	Intubation time: No significant difference between TAMS and OSD groups (62 seconds vs. 56 seconds, P = 0.30). Success rate: No significant difference, with all intubations successful. Complications: No significant difference (TAMS: 2 esophageal intubations; OSD: 4 esophageal intubations, P = 0.36). No technical errors were reported.
Meer, 2024 [15]	The intervention group used the SMASS symptom-checker for teletriage. Patients independently assessed their health status and complaints using the symptom-checker on a tablet PC. The symptom-checker used a computerized neural network to recommend appropriate triage levels and actions.	The control group (assumed) consisted of patients who were seen and assessed by routine medical staff in the walk-in clinic (WIC) and emergency department (ED) without using the symptom-checker.	• Safety of the symptom-checker (under-triage and over-triage rates), potentially hazardous under-triage. • Comparison of triage recommendations with medical experts.	No cases of potentially hazardous under-triage based on the consensus criterion. Four cases of potentially hazardous under-triage based on the majority criterion. The upper 95% confidence bound for the probability of a potentially hazardous under-triage was 0.3616%. Over-triage occurred in 17.69% of cases. Results suggest the symptom-checker is a safe triage tool.
Villa, 2020 [12]	Telemedicine conducted via "store-and-forward" method: Emergency physician takes images of skin lesions and sends them to a dermatologist for evaluation. Suspected diagnosis is made based on the transmitted images and clinical data.	Conventional treatment: Patients were seen and diagnosed directly by a dermatologist in the ED without telemedicine.	• Agreement between teledermatology diagnosis and clinical diagnosis. • Comparison of treatment times between telemedicine group and conventional group.	Primary outcome: 100% agreement between teledermatology diagnosis and subsequent in-person clinical diagnosis. Secondary outcome: Treatment time was significantly shorter in the telemedicine group (43 ± 38 minutes) compared to the conventional group (151 ± 71 minutes, p < 0.001). No significant changes in diagnoses after patient discharge between both groups.

Telemedicine interventions reduced re-consultation rates in some studies. For instance, one trial reported significantly lower re-consultation rates for telemedicine (14%) compared to control (26.5%) (p = 0.004) [14]. Telemedicine improved treatment adherence, with one study reporting higher adherence rates in the telemedicine group (97.5%) compared to the control group (92%) (p = 0.014) [14]. Patient satisfaction was generally high for telemedicine. One study found significantly higher satisfaction in the telemedicine group (90%) compared to the control group (37.5%) (p < 0.05) [14]. Healthcare providers generally reported positive experiences with telemedicine systems, although some expressed concerns about remote diagnosis accuracy [13]. Telemedicine reduced treatment times in non-critical settings. One trial reported significantly shorter treatment times in the telemedicine group (p < 0.001) [15]. Telemedicine systems demonstrated robust technical performance with minimal complications. One study observed no major complications, although minor delays in video and audio were noted [16]. One study observed a reduction in antibiotic prescriptions in the telemedicine group (5.9%) compared to the control group (17.6%), although this finding was not statistically significant (p = 0.125) [17].

Discussions

This systematic review explored the role of telemedicine in ED triage and patient care by analyzing six RCTs conducted in diverse settings. These studies investigated telemedicine interventions, such as tele-triage systems and real-time video consultations, focusing on outcomes like diagnostic accuracy, throughput times, re-consultation rates, treatment adherence, and satisfaction among patients and providers.

The findings suggest that telemedicine interventions are effective in improving diagnostic accuracy, reducing re-consultation rates, and enhancing treatment adherence. Improvements in patient satisfaction and reduced throughput times were particularly notable in non-emergency scenarios. However, limitations in study design, such as small sample sizes and challenges with blinding, may affect the generalizability of these results.

This review highlights the potential of telemedicine to enhance ED triage and patient care, especially in non-critical cases where remote consultations can efficiently reduce waiting times and improve patient outcomes. However, its effectiveness in complex emergency scenarios, such as those requiring hands-on interventions, remains less established, pointing to the need for further research in high-acuity settings.

The successful implementation of telemedicine in EDs depends on addressing key factors such as technological infrastructure, clinical workflows, and patient privacy. While telemedicine offers clear benefits in streamlining patient flow and enhancing resource allocation, its limitations in scenarios requiring immediate physical assessment warrant careful consideration.

This review also underscores several challenges, including the heterogeneity of telemedicine technologies and patient populations, which complicates direct comparisons between studies. The relatively small number of included studies and the lack of a quantitative synthesis limit the ability to draw comprehensive conclusions about its overall effectiveness.

Future research should prioritize larger and more diverse studies to evaluate the long-term impact and cost-efficiency of telemedicine interventions. Advanced technologies like artificial intelligence and machine learning should be explored to further improve diagnostic accuracy and decision-making in emergency scenarios. Additionally, assessing telemedicine’s applicability in high-acuity cases remains a crucial area for investigation.

## Conclusions

In conclusion, telemedicine offers significant potential for improving triage and patient care in EDs, particularly for non-critical cases. The results of this review suggest that telemedicine can enhance diagnostic accuracy, reduce re-consultation rates, improve treatment adherence, and streamline ED workflows, ultimately leading to better patient outcomes. However, further research is needed to evaluate its effectiveness in high-acuity emergency settings and to address the technological and logistical challenges associated with its implementation. Telemedicine should be viewed as a complementary tool to traditional in-person care, with the potential to transform ED operations when integrated effectively.

## References

[1] Sun R, Karaca Z, Wong HS (2006). Trends in hospital emergency department visits by age and payer, 2006-2015. Healthcare Cost and Utilization Project (HCUP) Statistical Briefs.

[2] Langabeer JR 2nd, Gonzalez M, Alqusairi D, Champagne-Langabeer T, Jackson A, Mikhail J, Persse D (2016). Telehealth-enabled emergency medical services program reduces ambulance transport to urban emergency departments. West J Emerg Med.

[3] Kelton DK, Szulewski A, Howes D (2018). Real-time video telemedicine applications in the emergency department: a scoping review of literature. CJEM.

[4] Witkowska-Zimny M, Nieradko-Iwanicka B (2022). Telemedicine in emergency medicine in the COVID-19 pandemic-experiences and prospects-a narrative review. Int J Environ Res Public Health.

[5] Sharifi Kia A, Rafizadeh M, Shahmoradi L (2022). Telemedicine in the emergency department: an overview of systematic reviews. Z Gesundh Wiss.

[6] Smith AC, Thomas E, Snoswell CL, Haydon H, Mehrotra A, Clemensen J, Caffery LJ (2020). Telehealth for global emergencies: implications for coronavirus disease 2019 (COVID-19). J Telemed Telecare.

[7] Kichloo A, Albosta M, Dettloff K (2020). Telemedicine, the current COVID-19 pandemic and the future: a narrative review and perspectives moving forward in the USA. Fam Med Community Health.

[8] Kruse CS, Krowski N, Rodriguez B, Tran L, Vela J, Brooks M (2017). Telehealth and patient satisfaction: a systematic review and narrative analysis. BMJ Open.

[9] Dorsey ER, Topol EJ (2020). Telemedicine 2020 and the next decade. Lancet.

[10] Page MJ, Moher D, Bossuyt PM (2021). PRISMA 2020 explanation and elaboration: updated guidance and exemplars for reporting systematic reviews. BMJ.

[11] Downs SH, Black N (1998). The feasibility of creating a checklist for the assessment of the methodological quality both of randomised and non-randomised studies of health care interventions. J Epidemiol Community Health.

[12] Villa L, Matz O, Olaciregui Dague K, Kluwig D, Rossaint R, Brokmann JC (2020). The assessment of dermatological emergencies in the emergency department via telemedicine is safe: a prospective pilot study. Intern Emerg Med.

[13] Brennan JA, Kealy JA, Gerardi LH, Shih R, Allegra J, Sannipoli L, Lutz D (1999). Telemedicine in the emergency department: a randomized controlled trial. J Telemed Telecare.

[14] Soltane HB, Lazrak I, Chelly S (2024). Place of telemedicine in the organization of emergency care: feasibility and benefits. BMC Emerg Med.

[15] Meer A, Rahm P, Schwendinger M, Vock M, Grunder B, Demurtas J, Rutishauser J (2024). A symptom-checker for adult patients visiting an interdisciplinary emergency care center and the safety of patient self-triage: real-life prospective evaluation. J Med Internet Res.

[16] Cho J, Chung HS, Choa M, Yoo SK, Kim J (2011). A pilot study of the tele-airway management system in a hospital emergency department. J Telemed Telecare.

[17] Accorsi TA, Moreira FT, Pedrotti CH (2022). Telemedicine diagnosis of acute respiratory tract infection patients is not inferior to face-to-face consultation: a randomized trial. Einstein (Sao Paulo).

